# Portable stroke detection devices: a systematic scoping review of prehospital applications

**DOI:** 10.1186/s12873-022-00663-z

**Published:** 2022-06-16

**Authors:** Susmita Chennareddy, Roshini Kalagara, Colton Smith, Stavros Matsoukas, Abhiraj Bhimani, John Liang, Steven Shapiro, Reade De Leacy, Maxim Mokin, Johanna T. Fifi, J Mocco, Christopher P. Kellner

**Affiliations:** 1 Department of Neurosurgery, Icahn School of Medicine at Mount Sinai, 1468 Madison Avenue, Annenberg Building, 8th Floor, New York, NY 10029 USA; 2 Department of Neurosurgery and Brain Repair, University of South Florida, Tampa, FL USA

**Keywords:** Stroke, Prehospital, Emergency medical services, Technology, Diagnosis

## Abstract

**Background:**

The worldwide burden of stroke remains high, with increasing time-to-treatment correlated with worse outcomes. Yet stroke subtype determination, most importantly between stroke/non-stroke and ischemic/hemorrhagic stroke, is not confirmed until hospital CT diagnosis, resulting in suboptimal prehospital triage and delayed treatment. In this study, we survey portable, non-invasive diagnostic technologies that could streamline triage by making this initial determination of stroke type, thereby reducing time-to-treatment.

**Methods:**

Following PRISMA guidelines, we performed a scoping review of portable stroke diagnostic devices. The search was executed in PubMed and Scopus, and all studies testing technology for the detection of stroke or intracranial hemorrhage were eligible for inclusion. Extracted data included type of technology, location, feasibility, time to results, and diagnostic accuracy.

**Results:**

After a screening of 296 studies, 16 papers were selected for inclusion. Studied devices utilized various types of diagnostic technology, including near-infrared spectroscopy (6), ultrasound (4), electroencephalography (4), microwave technology (1), and volumetric impedance spectroscopy (1). Three devices were tested prior to hospital arrival, 6 were tested in the emergency department, and 7 were tested in unspecified hospital settings. Median measurement time was 3 minutes (IQR: 3 minutes to 5.6 minutes). Several technologies showed high diagnostic accuracy in severe stroke and intracranial hematoma detection.

**Conclusion:**

Numerous emerging portable technologies have been reported to detect and stratify stroke to potentially improve prehospital triage. However, the majority of these current technologies are still in development and utilize a variety of accuracy metrics, making inter-technology comparisons difficult. Standardizing evaluation of diagnostic accuracy may be helpful in further optimizing portable stroke detection technology for clinical use.

**Supplementary Information:**

The online version contains supplementary material available at 10.1186/s12873-022-00663-z.

## Background

Stroke is a severe medical emergency and a leading cause of morbidity and mortality worldwide, [1] causing approximately 1 in every 19 deaths in the United States alone [2].

Advances in stroke treatment, particularly endovascular therapy (EVT), have been shown to be highly effective in improving functional outcome in patients with emergent large vessel occlusion (LVO) [3–6]. However, EVT outcomes are time-dependent, with every 15-minute decrease in stroke onset to EVT arterial puncture associated with an increased chance of independent ambulation (absolute increase 1.14% [95% CI: 0.75–1.53%]) and modified Rankin Scale 0–2 (absolute increase 0.91% [95% CI, 0.45–1.36%]) at discharge [6, 7]. With only 10% of stroke centers capable of providing EVT, [8, 9] delayed or inaccurate prehospital diagnosis may increase need for interhospital transfer, associated with a 116-minute average time-delay, thereby increasing time-to-revascularization for LVO patients and placing undue burden on stroke centers tasked with treating incorrectly diagnosed stroke mimics [10, 11]. Finally, emerging evidence that timely treatment of intracranial hemorrhage is associated with improved clinical outcomes further suggests that accurate early recognition of different stroke types could have substantial effect on recovery after stroke [12].

Currently, prehospital diagnosis of stroke relies on stroke triage scales, and the results of these assessments provide the basis for emergency medical service (EMS) transport decisions. The diagnostic accuracy of such scales in detecting large vessel occlusions, however, is low, ranging from 55 to 89% sensitivity, 40–92% specificity, and 0.73–0.78 area under the ROC curve (AUC) [13–19]. This variability in accuracy may be the result of varying stroke types, severity, and presenting symptoms, as well as inter-state protocol differences, resulting in misdiagnosis of stroke types and, as consequence, selection of less appropriate hospital types for initial stroke admission and increased necessity for interhospital transfer [20–23].

Thus, the integration of portable diagnostic technology in standard prehospital stroke care may improve triage and play an important role in reducing transportation time to the appropriate hospital, allowing for increased treatment efficiency and improved functional outcomes for stroke patients. Therefore, the purpose of this study was to conduct a systematic scoping review intended to (1) identify and characterize novel portable technologies with the potential to diagnose stroke in the prehospital setting; (2) report diagnostic accuracy and feasibility of use of identified technologies; and (3) assess the quality of any included studies.

## Methods

A systematic scoping review methodology was selected to identify available diagnostic accuracy and feasibility of use data for novel portable stroke detection devices and to identify any knowledge gaps in this emerging field, as recommended by previous literature [24] and an experienced librarian. Due to the scoping nature of this study, its protocol has not been prospectively registered. This study utilizes the methodology framework described by Arksey and O’Malley [25] and follows the Preferred Reporting Items for Systematic Reviews and Meta-Analyses Extension for Scoping Reviews (PRISMA-ScR) reporting guidelines [26].

### Review inclusion criteria

Any study reporting a portable, non-invasive technology or device with potential for prehospital detection of stroke or intracranial hemorrhage was eligible for inclusion. Studies were required to report diagnostic accuracy results (including, but not limited to, specificity, sensitivity, and/or area under the curve) after testing on patient cohorts experiencing ischemic stroke, hemorrhagic stroke, or intracranial hemorrhage in clinical settings for inclusion.

### Review exclusion criteria

Studies were excluded if they reported on technology that requires use of specialized vehicles (ex. mobile stroke units using mobile CT technology) and could not be translated into handheld, easily transported devices. Studies solely testing computer algorithm-based stroke detection without other portable technology applications were also excluded, as were studies that tested technology only on phantom individuals rather than real world patients. Case studies, studies not written in English, and studies without available full texts were also excluded.

### Search strategy

A search strategy was developed alongside an experienced librarian following a series of preliminary searches identifying studies and key terms relevant to the study questions. Final search strategies were applied to PubMed and Scopus until January 2021 and are found in Additional file 1: Appendix 1. Similar articles and articles cited by included studies were also retrieved.

### Study selection

Retrieved articles were imported into EndNote X9 citation software, and following de-duplication, two reviewers (S.C. and R.K.) screened title/abstracts and retained full-text studies based on identified inclusion and exclusion criteria to select the final list of included studies. Any concerns about inclusion were resolved by discussion with author C.P.K.

### Data extraction

Data were extracted independently by two reviewers (S.C. and R.K.). Any discrepancies were resolved by discussion. A data extraction tool was developed using previous reporting frameworks and published reviews as guidance [27, 28].Included fields consisted of general study design as well as more in-depth characterization of technologies, diagnostic accuracy, and feasibility of use. The extraction tool also included a field for cost of application, but as no identified studies reported cost data, that field is not reported here.

### Data synthesis

No further data analysis was undertaken due to the lack of standardized reporting for diagnostic accuracy in this field and the scoping nature of this review.

### Quality assessment

The quality of included studies was assessed independently by two reviewers (S.C. and R.K.) using the Quality Assessment of Diagnostic Accuracy Studies-2 (QUADAS-2) tool, [29] with any discrepancies were resolved by discussion. The QUADAS-2 tool reviews risk of bias and applicability specific to diagnostic accuracy reporting through four domains, including patient selection, index test, reference standard, and flow and timing. Risk of bias is reported in each domain as high, low, or unclear.

## Results

**Fig. 1:**
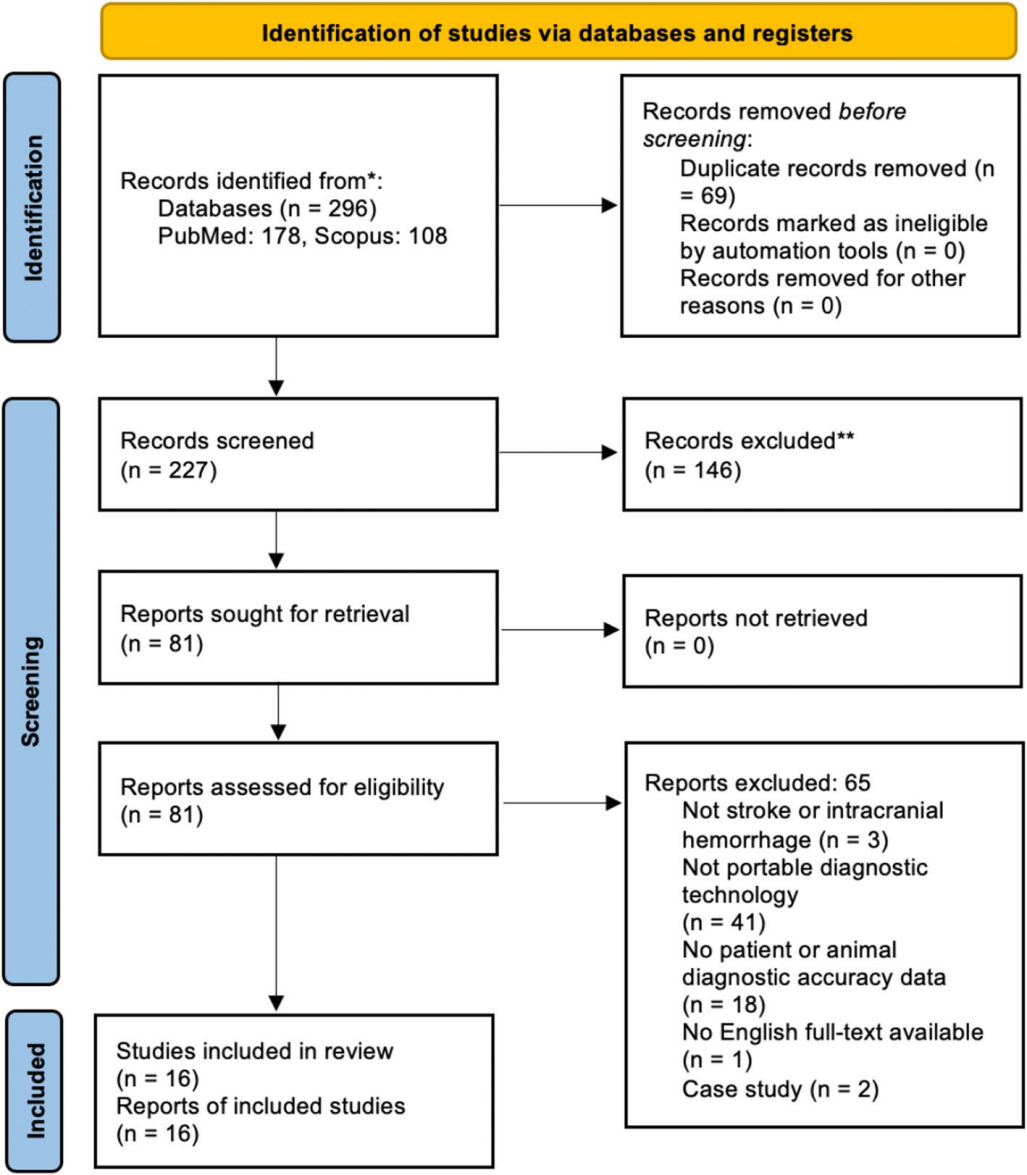
PRISMA flow diagram for study inclusion

Our review identified 227 studies after application of the search strategy and deduplication. A total of 81 studies were selected for full text screening, during which 65 studies were excluded for the reasons listed in Fig. 1 based on review criteria. Sixteen studies were included in the final analysis. Assessed risk of bias of each included study is shown in Table 1.

**Table 1:** QUADAS-2 tool for risk of bias assessment for included studies

INCLUDED STUDIES	PATIENT SELECTION	INDEX TEST	REFERENCE STANDARD	FLOW AND TIMING
Michelson, et al.	Low	Low	High	Low
Wilkinson, et al.	Low	Low	Low	High
Herzberg, et al.	Low	Low	Low	Low
Schlachetzki, et al.	Low	Low	Low	Low
Antipova, et al.	Low	Low	Low	Low
Erani, et al.	Low	Low	Unclear	Unclear
Kellner, et al.	Low	Low	Low	Unclear
Thorpe, et al.	High	High	Low	High
Sergot, et al.	High	Low	Low	Low
Persson, et al.	Unclear	Low	Low	High
Robertson, et al.	Low	Low	Low	Low
Liang, et al.	Low	Low	Low	Low
Xu, et al.	Low	Low	Low	Low
Peters, et al.	High	Low	Low	Low
Yuksen, et al.	Low	Low	Low	Unclear
Kontojannis, et al.	Low	Unclear	Low	Low

Characteristics of the included studies are summarized in Table 2. Six of the included studies described near-infrared spectroscopy (NIRS) technology, 4 tested ultrasound technologies, 4 used diagnostic electroencephalography (EEG) scanning, 1 studied microwave technology, and 1 used a volumetric impedance phase shift spectroscopy (VIPS) device. The majority of studies were conducted in North America (7) and Europe (6), with the majority of devices being tested in the United States (6). Three of the studies described prehospital testing of devices (either on-site or during hospital transport). Six devices were tested in emergency departments (EDs), and the remaining 7 were tested in hospital or stroke center settings. The majority of studies required trained personnel or clinicians for device use, while output was often computer or device-generated. All identified studies described non-invasive, portable devices with potential prehospital applications. Median time for measurement was 3 minutes (IQR: 3 minutes to 5.6 minutes). No cost data were available in any study.

**Table 2:** Characterization of Included Studies

No.	Author	Year	N	Country	Technology Type	Location of Use	Expertise Needed for Device Use	Expertise Needed for Output
1	Michelson	2015	183	USA	EEG	ED	Not reported	EEG technologist, computer-based
2	Wilkinson	2020	25	Canada	EEG	Stroke Unit	Not reported	Computer-based
3	Herzberg	2014	102	Germany	Ultrasound	On-site, ambulance	TCCS-experienced neurologist	Experienced sonographer
4	Schlachetzki	2012	113	Germany	Ultrasound	On-site, ambulance	Neuro-sonography-certified clinicians	Not reported
5	Antipova	2020	107	UK	Ultrasound	Hospital	Experienced neurologist, sonographer	Neurologist, radiologist, stroke physician
6	Erani	2020	100	USA	EEG	ED	Not reported	Computer-based
7	Kellner	2018	248	USA	VIPS	Stroke center	Trained personnel	Computer-based
8	Thorpe	2018	66	USA	Ultrasound	Stroke center	Trained technician	Computer-based
9	Sergot	2021	109	USA	EEG	ED	Users after 1-hr training	Computer-based
10	Persson	2014	20 (S1) 90 (S2)	Sweden	Microwave	Hospital	Engineering, neuro-physiology, nursing staff	Computer-based
11	Robertson	2010	365	USA, India	NIRS	ED/Trauma	Trained operators after ½ day training	Computer-based, read by clinicians
12	Liang	2018	102	China	NIRS	Hospital	Trained operators	Computer-based
13	Xu	2017	85	China	NIRS	Hospital	Trained operators after ½ day training	Computer-based
14	Peters	2017	25	Netherlands	NIRS	Helicopter EMS	Trained HEMS physicians	Trained HEMS physicians
15	Yuksen	2020	47	Thailand	NIRS	ED	Trained emergency physician	Not reported
16	Kontojannis	2019	205	UK	NIRS	ED	Trained operators after ½ day training	Not reported

### Stroke detection

Ten devices were used for the purpose of stroke detection, including differentiating strokes from stroke mimics or healthy controls, detecting middle cerebral artery (MCA) occlusion, detecting or differentiating severe stroke or large vessel occlusions (LVOs), and differentiating between stroke types (Table 3).

**Table 3:** Characterization of included portable stroke technologies and associated diagnostic accuracy metrics

No	Author	Year	Technology Name	Purpose	Specificity	Sensitivity	AUC	PPV	NPV	Comparator	Time to results
1	Michelson	2015	Hand-held EEG	Stroke detection	50.40% (43.0–58.7%)	91.7% (80.5–96.7%)	–	39.6% (30.6–49.4%)	94.40% (85.7–98.2%)	CT/MRI	10 min
2	Wilkinson	2020	Muse	Stroke detection	86%	63%	–	–	–	CT, MRI	3 min
3	Herzberg	2014	SonoSite Micromaxx; Philips CX50	Stroke detection	48% (29–67%)	94% (86–98%)	–	82% (72–89%)	77% (52–93%)	CT, CTA, MRA	5.6 min
4	Schlachetzki	2012	SonoSite Micromaxx; Philips CX50	MCA occlusion	98% (92.89–99.97%)	90% (55.5–99.75%)	–	90% (55.5–99.75%)	98% (92.89–99.97%)	CT, MRA, neuro-Sonography	5.6 min
5	Antipova	2020	SonoSite M-Turbo; Philips Sparq; Philips CX50	LVO detection	97%	55%	0.93 (0.865–0.996)	75%	93%	CT	20 min
				ICH detection	99%	63%	0.912 (0.829–0.996)	91%	92%		
6	Erani	2020	Quick-20	LVO detection	80%	41%	68.9	–	–	–	3 min
				Acute stroke/TIA	80%	65%	78.2				
7	Kellner	2018	Cerebro-tech Visor	Severe vs. small stroke	92% (75–99%)	93% (83–98%)	0.93 (0.85–0.97)	96% (88–99%)	86% (70–94%)	Triage scales, imaging	30 sec
				Severe stroke detection	87% (81–92%)	93% (83–98%)	0.93 (0.89–0.96)	70% (61–77%)	98% (94–99%)		
8	Thorpe	2018	2 MHz hand-held ultrasound	LVO detection	82% (VAI)	82% (VAI)	0.88 (VAI)	–	–	CTA	30 sec
					88% (VCI)	91% (VCI)	0.94 (VCI)				
9	Sergot	2021	PLD	LVO detection	80% (77–83%)	80% (74–85%)	–	–	–	CTA	4.6 min
10	Persson	2014	2 helmet-design prototypes	ICH/IS (S1)	–	–	0.88	–	–	Clinical, radiography	–
				ICH/IS (S2)			0.85				
							0.87				
				ICH/Control							
11	Robertson	2010	Infra-scanner	Any intracranial hemorrhage detection	90.70% (86.4–93.7%)	68.70% (58.3–77.6%)	–	72.50% (62.0–81.1%)	89% (84.6–92.3%)	CT	< 2 min
				Detection in Infrascanner limits	90.70% (86.4–93.7%)	88% (74.9–95.0%)	–	63.70% (51.2–74.7%)	97.6% (94.6–99.0%)		
12	Liang	2018	Infra-scanner 2000	Intracranial hematoma detection in Infrascanner limits	93.6% (85–97.6%)	100% (82.8–100%)	–	82.8% (63.5–93.5%)	100% (93.8–100%)	CT	< 3 min
13	Xu	2017	Infra-scanner 2000	Intracranial hematoma detection in Infrascanner limits	92.50% (78.5–98%)	95.60% (83.6–99.2%)	0.97	93.50% (81.1–98.3%)	94.90% (81.4–99.1%)	CT, MRI	< 3 min
14	Peters	2017	Infra-scanner 2000	Intracranial hematoma detection	78.60%	93.30%	–	–	–	CT	4 min
15	Yuksen	2020	Infra-scanner 2000	Intracranial hematoma detection	44.4% (35.8–44.40%)	100% (71.90–100%)	0.722	35.5% (25.5–35.5%)	100% (80.7–100%)	CT	3 min
16	Kontojannis	2019	Infra-scanner 2000	Any intracranial hematoma detection	50.43% (41.03–59.80%)	75% (64.63–83.62%)	–	53.23% (47.76–58.62%)	72.84% (64.16–80.07%)	CT	3.74 min
				Intracranial hematoma detection in Infrascanner limits	48.73% (40.71–56.80%)	89.36% (76.90–96.45%)	–	34.15% (30.2–38.33)	93.90% (86.88–97.28)		

Three devices were used to differentiate stroke from stroke mimics or healthy controls, including two studies testing EEG devices and one study using ultrasound technology.

Michelson, et al. [30] presented a US multicenter study analyzing the diagnostic accuracy of a hand-held EEG device (BrainScope Co., Inc.) in detecting stroke in 183 patients (31 ischemic stroke, 17 hemorrhagic stroke, 135 stroke mimic confirmed by CT/MRI) presenting to the ED. Ten minutes of EEG data were recorded, and data analysis was done through a derived Structural Brain Injury Index (SBII) algorithm. Stroke detection had a sensitivity of 91.7% and specificity to stroke mimic of 50.4%. The study reported a > 90% accuracy in detecting ischemic and hemorrhagic stroke, with 80% sensitivity to CT- MRI+ ischemic stroke. Authors noted that many included patients may have experienced prior strokes or transient ischemic attacks, which may disrupt baseline EEG recordings, and thus clinical applicability may benefit from inclusion of a control group.

Wilkinson, et al. [31] discussed a small single-center study that used the Muse (InteraXon Inc., Toronto, ON) electroencephalography system to detect stroke severity in 25 patients presenting to a university hospital in Canada. Patients were assessed an average of 3.71 days after stroke onset. The authors noted an increase in delta/alpha ratio and (delta+theta)/(alpha+beta) ratio in ischemic stroke patients with increased severity, as well as a low frequency decrease and high frequency increase in pairwise-derived Brain Symmetry Index in stroke patients compared to controls. The device was able to differentiate moderate/severe stroke from small strokes and controls (as diagnosed by CT, MRI, and stroke scale assessment) with a sensitivity of 63% and specificity of 86%. Scans were recorded for 3 minutes in eyes-open and -closed states. Feasibility of prehospital use may benefit from automatability of EEG interpretation but results from this study are limited by small sample size and may be less applicable to more acute stroke cases.

Herzberg, et al. [32] described a single-site study in Germany that used two portable ultrasound machines capable of transcranial color-coded sonography (TCCS), SonoSite Micromaxx (SonoSite Inc., Bothell, Wash., USA) and Philips CX50 (Philips Ultrasound, Bothell, Wash., USA, to detect stroke in 102 patients on-site or during ambulance transport. Examinations were performed by a TCCS-experienced neurologist, lasted 5.6 minutes on average, and were reviewed by a certified sonographer. Compared with in-hospital CT, CTA, or MRA imaging, TCCS was able to differentiate stroke from stroke mimics with 94% sensitivity and 48% specificity. Authors note increased diagnostic accuracy in detection of MCA occlusion with addition of TCCS examination.

### Detection of MCA occlusion

One study, Schlachetzki, et al. [33], also described the use of SonoSite Micromaxx and Philips CX50 in the prehospital setting for the detection of middle cerebral artery (MCA) occlusion on-site or during ambulance transit in Germany. Contrast agent microbubbles were added in some cases and improved results in cases with inadequate temporal bone windows. The device was used to examine 113 enrolled patients with symptoms of acute ischemic stroke, and examinations were performed by board-certified stroke neurologists or a senior resident with neurosonography certification. Based on CT angiography, MR angiography, and in-house neurosonography, 9 out of 10 MCA occlusions were identified correctly, while patent MCAs were correctly identified in 75 out of 76 cases. Sensitivity and specificity were reported to be 90 and 98%, respectively. The average time needed to perform the TCCS scan was 5.6 minutes and did not delay prehospital management. Prehospital use may be partially limited by the expertise and training needed to perform ultrasound examinations.

### Detection of LVO/severe stroke

Five studies used devices to detect LVO or severe stroke, with 2 studies testing ultrasound technologies, 2 testing EEG devices, and 1 using a VIPS device. One study [34] also reported diagnostic accuracy of ICH detection with the same ultrasound device, and another study [35] reported higher diagnostic accuracy in detecting any stroke/TIA than detecting LVO alone with an EEG device.

Antipova, et al. [34] was a single-center study that performed non-contrast TCCS using a SonoSite M-Turbo Point-of-Care ultrasound machine, Philips Sparq, or Philips CX50 ultrasound on 107 patients presenting to a general hospital in the UK with acute stroke symptoms. Imaging was performed by either an experienced sonographer or a neurologist with several years of experience in transcranial ultrasonography; results were interpreted by the neurology team. Compared to reference CT imaging, large vessel occlusion (LVO) was detected correctly in 7/13 suspected LVO patients (4 cases were missed and 2 had inadequate temporal windows). ICH was correctly identified in 10/18 suspected patients. Use of transcranial ultrasound improved ICH detection from clinical assessment alone by 10% (57% increased sensitivity with no changes in specificity). LVO detection based on clinical assessment and transcranial ultrasound showed a sensitivity of 55% and specificity of 97% with a 6% overall improvement in detection. Time needed scan completion ranged from 7 to 49 minutes, with a median of 20 minutes. The study presents a triage model combining both clinical assessment and ultrasound, which could reduce need for interhospital transfers. However, clinical use may be limited by time required for scan completion, expertise requirements for operation, and temporal window insufficiencies.

Erani, et al. [35] tested the Quick-20 (Cognionics, Inc., San Diego, CA; Fig. [A]) electroencephalography system in 100 ED patients with suspected acute stroke, including 43 patients with ischemic stroke, 7 with ICH, and 13 with TIA. While EEG variables alone resulted in a 65% sensitivity at 80% specificity with an AUC of 78.2 in detecting acute stroke/TIA or not, combined clinical and EEG measures with deep learning increased sensitivity to 79% with an AUC of 87.8. Similarly, EEG alone had a 41% sensitivity and AUC of 68.9 in identifying LVO or not, while the combination increased sensitivity to 76% and AUC to 86.4. Overall, authors noted the diagnostic accuracy of clinical data and EEG combined was better than either alone at detecting acute stroke/TIA or LVO. Scans were recorded for 3 minutes.

Kellner, et al. [19] tested a volumetric impedance phase shift spectroscopy (VIPS) visor device manufactured by Cerebrotech in 248 subjects including 41 stroke code patients, 79 healthy volunteers, and 128 patients presenting to a comprehensive stroke center with varied neurological pathologies. The diagnostic accuracy of the device in differentiating severe stroke from minor stroke and severe stroke from all other pathologies was evaluated. Severe stroke was defined as emergent large vessel occlusion (ELVO), severe intracranial stenosis with National Institutes of Health Stroke Scale (NIHSS) scores ≥6, ICH ≥60 mL, and large territorial strokes. Authors noted differences in detected mean bioimpedance asymmetry (MBA) between severe stroke, mild stroke, and control patients. The device performed with a specificity of 92% and sensitivity of 93% in differentiating severe stroke from small stroke and a specificity of 87% and sensitivity of 93% in differentiating severe stroke subjects from all other cases. Three scans were taken in succession by study personnel trained by Cerebrotech in device usage, and total scan time was approximately 30 seconds. Future studies validating results from this preliminary analysis are needed.

Thorpe, et al. [36] studied the diagnostic accuracy of two transcranial doppler (TCD) ultrasound metrics, Velocity Asymmetry Index (VAI) and Velocity Curvature Index (VCI), with 2-MHz handheld ultrasound probes to detect LVO in 66 subjects (33 CTA-confirmed LVO, 33 in-hospital controls). TCD scans were performed by trained technicians and were recorded over 30 seconds. Means of both VAI and VCI were found to be greater in control subjects relative to LVO. Sensitivities and specificities for VAI and VCI metrics varied slightly based on specified statistical thresholds, yet the authors noted the superiority of the VCI metric compared to VAI.

Sergot, et al. and the EDGAR Study Group [37] conducted a multicenter study testing the PLD (Forest Devices, Inc., Pittsburgh, PA) in detecting LVO in a cohort of 109 patients (25 LVO, 38 non-LVO ischemic, 14 hemorrhagic, 32 mimics confirmed by CTA). The device demonstrated an 80% sensitivity and specificity in LVO discrimination. Scans were applied by users after a 1-hour training and required a median of 4.6 minutes to conduct. Authors noted that PLD was obtained after imaging and intravenous thrombolysis in most cases, which may have impacted PLD accuracy, implicating the need for validation studies prior to clinical use.

### Stroke characterization

Persson, et al. [38] performed two proof-of-principle clinical studies testing two different microwave-based prototype devices with machine-learning-derived data analysis differentiating ischemic stroke from hemorrhagic stroke. In the first study, neurophysiology and engineering staff tested the first prototype helmet device on 20 acute stroke patients (9 ischemic, 11 hemorrhagic). When the detector was set to diagnose all ICH patients, 7/11 ischemic stroke (IS) patients were able to be differentiated from ICH patients. Diagnostic accuracy, measured by AUC, was determined to be 0.88. In the second study, nursing staff tested the second prototype on 25 patients (15 IS, 10 ICH). When the detector was set to diagnose all 10 ICH patients, 14/15 IS patients were able to be correctly differentiated from ICH patients. Authors noted an AUC of 0.85 in differentiating ICH and IS patients and an AUC of 0.87 in differentiating ICH patients from healthy controls. Scan time was not reported. Larger cohort studies likely need to be done to better characterize the potential of microwave-based prehospital stroke diagnosis.

### Detection of intracranial hemorrhage

There were six studies that tested an Infrascanner NIRS device to detect intracranial hemorrhage (Table 3).

Robertson, et al. [39] conducted a multicenter study evaluating the diagnostic accuracy of the near-infrared (NIR)-based Infrascanner device (InfraScan, Inc.) in detecting intracranial traumatic hematomas in 365 patients (269 controls and 96 patients with intracranial hemorrhage). Scanning was completed by study personnel who completed a half-day NIR device training. Device performance was affected by type and size of hemorrhage. Authors reported a 68.70% sensitivity and 90.70% specificity in detecting any intracranial hemorrhage and 88% sensitivity and 90.70% specificity in detecting hematomas with volume > 3.5 mL and distance < 2.5 cm from brain surface, the detection limits of the device. The entire scan required less than 2 minutes to complete and was performed within 40 minutes of the comparator CT scan. Clinical use of this device may be limited by lack of diagnostic accuracy data in patients with scalp lacerations or head injuries.

The remaining five studies (Liang, et al. [40]; Xu, et al. [41]; Peters, et al. [42]; Yuksen, et al. [43]; and Kontojannis, et al. [44]) were smaller single-center studies in China, the Netherlands, Thailand, and the UK that used a newer Infrascanner Model 2000 device (InfraScan, Inc., Philadelphia, PA, USA). Sensitivity for intracranial hematoma detection ranged from 75 to 100% and specificity ranged from 44.4 to 93.6%. Specification of hematoma identification within Infrascanner detection limits (volume > 3.5 mL and depth < 2.5 cm from brain surface) improved sensitivity to 89.36–100%. Scans were all conducted by trained operators and required 3–4 minutes for completion.

Differences in diagnostic accuracy values may be explained by size of hematomas (sensitivity was reported to be higher in patients with larger bleeds [39]), which may indicate the necessity of future studies testing the device in larger patient cohorts and stratifying diagnostic accuracy by hemorrhage size or type. Differences could also be explained by selection of different patient populations (with differences in skin and hair color potentially altering device sensitivity), differences in operator training protocols, and differences in study setting (two studies reported incomplete scans that did not assess all brain regions due to difficulty accessing those regions in prehospital or emergency environments).

## Discussion

Many emerging portable stroke technologies can detect and differentiate stroke subtypes and severity. Three studies tested devices differentiating stroke from stroke mimics and controls, [30–32] one detected MCA occlusion, [33] five detected LVO and severe stroke, [19, 34–37], one differentiated between ischemic and hemorrhagic stroke, [38] and six detected intracranial hematoma [39–44].

While current prehospital stroke diagnosis relies on stroke triage scales, diagnostic accuracy for EMS stroke or TIA identification with triage scales alone remains low (positive predictive value 34.3% [95% CI: 33.7–35.0], sensitivity 64.0% [95% CI: 63.0–64.9]) [45]. Many of the selected studies presented technologies that were capable of detecting and stratifying stroke with higher diagnostic accuracy than clinical assessment or triage scales alone. Integration of technology with clinical assessment may enhance stroke detection and reduce false-positive stroke diagnosis in the prehospital setting, which could allow EMS to make more informed decisions about bypass transportation to EVT-capable comprehensive stroke centers, thereby reducing time-to-treatment and improving clinical outcomes for severe stroke patients.

All the included studies were designed in a manner that allowed them to calculate diagnostic accuracy, and most described some level of blinding, with either device operators blinded to reference standard findings or clinicians blinded to device findings. Blinding to clinical presentation was not possible in any study, and time between device scanning and reference test varied by study, increasing risk of bias. Only three studies were conducted in prehospital settings, [32, 33, 42] which may limit the generalizability of studies conducted in ED or hospital settings. Prehospital applications may also be limited by training requirements for device use. Only a few identified articles were larger multicenter studies; thus, validation studies with larger sample sizes may be needed prior to clinical application.

Previous studies have reported similar categories of portable stroke detection technology. Walsh, et al. [46] reported ten devices in development, most of which had not been tested nor published in peer-reviewed journals. Martinez-Gutierrez, et al. [47] presents similar devices in development, along with mobile stroke unit technology and stroke scale applications, both of which were excluded here. Lumley, et al. [27] discussed a large variety of prehospital diagnostic technologies, including blood biomarkers and telemedicine technology, but only included two studies involving imaging technology [48, 49]. Shahrestani, et al. [50] reviewed stroke point-of-care technologies tested on human subjects or phantom head models, several of which are presented here; this review, however, focuses on devices with available human-centered diagnostic accuracy metrics. Several other studies [51–61] identified by search strategy were not presented here due lack of complete diagnostic accuracy data, but may prove to be promising technologies for prehospital stroke detection in the future.

While this paper provides a systematic approach to identifying emerging portable stroke detection devices, it also has several limitations. The systematic nature of this paper limits its scope in that it excludes studies that are unpublished, not peer-reviewed, or lacking diagnostic accuracy data. Thus, emerging technologies in preliminary stages of testing, without published diagnostic accuracy in human subjects, were not identified. Furthermore, this study does include technologies with intended prehospital use that have not yet been tested in prehospital settings, many of which may require validation in prehospital settings prior to clinical use. Finally, the protocol for this systematic scoping review was not registered, and due to the nature of the included literature, analyses were not performed in this study.

### Future directions

In the future, diagnostic accuracy reporting for portable stroke detection devices should be standardized. Differences in study design and study populations compounded with differences in reported accuracy metrics and protocol make inter-technology comparisons difficult. More comprehensive reporting of reference imaging standards, average imaging time and time to imaging results, device invasiveness and portability, expertise requirements for device use and result analysis, device training protocols, and eventually expected device costs would better inform clinical applications of each device and reduce study bias. Furthermore, patient populations assessed with device technology were widely varied in the included studies; clear reporting of patient populations, characteristics, and clinical presentations would clarify potential uses and settings for technology implementation. While early device validation in-hospital settings provide a valuable method to assess a preliminary patient population within reasonable limits of time and personnel, performing validation studies of early promising devices with larger, multicenter studies in prehospital settings would better establish the value and efficacy of current technologies. Finally, few studies compared diagnostic accuracy of device use with accuracy of clinical assessment alone; such comparisons may better inform the value of these devices in prehospital triage beyond current standards of practice.

Impact of portable stroke technology on reducing time-to-treatment, improving patient outcomes, and reducing healthcare costs should also be investigated by modeling the implementation of reviewed technologies at various points of care in pre-hospital and hospital settings. By integrating literature-based estimates of functional outcome benefits and cost reduction relative to time reduced by technology integration, such a model may inform future methods to optimize prehospital triage and EMS bypass policies.

### Key recommendations

The results of this review should be considered when designing future detection technologies and reporting on these trials. To begin, we recommend standardization in reporting of diagnostic accuracy statistics. Future analyses should report, at minimum, the following variables: specificity, sensitivity, ROC curves, positive predictive value (PPV), and negative predictive value (NPV). Moreover, the comparator standard should also be clearly indicated. Reporting these values will allow for meaningful and complete comparisons to be drawn between devices and will be important in guiding future research.

The patient populations assessed by technologies should be clearly defined. Most included studies reported eligibility criteria, as well as settings and dates of participant enrollment. The method of patient enrollment (randomized, consecutive, etc.) should be stated. Importantly, included population characteristics should be described in detail. Beyond demographic characteristics, studies should include the clinical presentation and impression of suspected stroke as well as results of any conducted pre-hospital or neurological triage scales. Such reporting would allow for better comparison between reported devices and potential applications.

In addition, there should be standard operating characteristics that are reported in future studies. This information should clearly indicate the following: level of training required for device use, identification of the individual operating the device and any relevant qualifications (physician, trained research technician, etc.), setting and location of technology use, timing of device use within the chain of stroke care, and time to scan completion. The method of scan analysis should also be reported, including description of device results and any rationale or algorithms related to the final determination of stroke identification. Additionally, the degree of blinding in research protocol, for device operators, any individuals assessing device results, and individuals assessing the reference test, should be clearly stated. The aforementioned data is essential to reproducibility, quality assessment, and potential for prehospital use and, while the several studies did include this information in some capacity, it should be clearly stated in the methods section of all future literature analyzing novel devices.

Furthermore, we propose that ease of use and applicability must be prioritized when designing future interventions. As noted above, some of the included devices required highly specialized operators, limiting the potential to make these devices universal. Devices that require minimal training for use and interpretation will be the most feasible for incorporation into prehospital personnel training for use in the prehospital environment. Few studies noted the additional benefit of devices on diagnostic accuracy of clinical or prehospital triage scales alone. Importantly, the applicability and value of stroke detection devices in a real-world setting would be greatly informed by such comparisons, which should be considered in the design process of future studies of device-based diagnostic accuracy.

Finally, although all the devices included in this study were portable, they were tested in various contexts, from prehospital settings to specialized hospital units. Early device validation in hospital settings may be more feasible for device assessment in a standard patient population within reasonable limits of time and expertise. However, future RCTs may benefit from analyzing device use in multiple settings, particularly after initial validation, and should report this setting-specific diagnostic data. This future literature can hopefully lead to the development of stroke detection devices that are specialized for use in different parts of the care chain, from emergency situations in the prehospital environment to daily monitoring during in-patient care.

As highlighted by this study, there is a current lack of standardization in reporting of diagnostic accuracy data of portable stroke detection devices. In 2015, Cohen et al. developed a checklist, titled “Standards for Reporting of Diagnostic Accuracy Studies” (STARD) that may be used to ensure sufficient reporting of data in a range of fields [62]. Subsequently, field-specific STARD guidelines have emerged for the research of various disease states, including dementia [63] and various infectious processes, [64] among others [65]. In line with this past literature, we recommend the development of STARD-PSD guidelines, focused on creating standards for portable stroke device technology.

## Conclusion

While numerous portable, non-invasive technologies have emerged as promising tools for the detection and stratification of stroke subtypes, most are still in development and have not yet been tested in large multicenter or prehospital settings. Moreover, included studies report a variety of study designs, study populations, and diagnostic accuracy metrics, making inter- technology and inter-device comparisons particularly difficult. Standardized reporting of diagnostic accuracy metrics, requirements for device training and use, studied patient populations and characteristics, and comparison of device accuracy with that of clinical assessment alone may better inform the value of portable stroke detection technology in prehospital triage.

## Supplementary Information

**Additional file 1.**

## Data Availability

The authors declare that all data supporting the findings of this study are available within the article and its supplementary files.

## Abbreviations

EVT: Endovascular therapy
LVO: Large vessel occlusion
EMS: Emergency medical services
AUC: Area under the curve (ROC curve)
CSC: Comprehensive stroke center
NIRS: Near-infrared spectroscopy
EEG: Electroencephalography
VIPS: Volumetric impedance phase shift spectroscopy
TCCS: Transcranial color-coded sonography
MCA: Middle cerebral artery
TCD: Transcranial doppler
IS: Ischemic stroke
ICH: Intracerebral hemorrhage
PPV: Positive Predictive Value
NPV: Negative Predictive Value
