# Supplementary material for: Portable stroke detection devices: a systematic scoping review of prehospital applications
Source: BMC Emerg Med. 2022 Jun 16;22:111. doi: 10.1186/s12873-022-00663-z (PMC9204948; doi:10.1186/s12873-022-00663-z)
Supplement: Supplementary file 1 — Additional file 1. [file 12873_2022_663_MOESM1_ESM.docx]

**Appendix: Search Terms**

PubMed

prehospital AND (stroke OR cerebrovascular accident OR cerebrovascular accidents) AND (technology or technologies OR spectroscopy OR transcranial doppler OR radiofrequency OR near-infrared OR microwaves OR accelerometers OR electroencephalography OR device OR devices)

Scopus

( TITLE-ABS-KEY ( prehospital ) AND TITLE-ABS-KEY ( stroke OR "cerebrovascular accident" OR "cerebrovascular accidents" ) AND TITLE-ABS-KEY ( technology OR technologies OR spectroscopy OR doppler OR radiofrequency OR infrared OR microwaves OR accelerometers OR electroencephalography OR device OR devices ) )
